# Supplementary material for: The gut efflux pump MRP-1 exports oxidized glutathione as a danger signal that stimulates behavioral immunity and aversive learning
Source: Commun Biol. 2022 May 5;5:422. doi: 10.1038/s42003-022-03381-1 (PMC9072357; doi:10.1038/s42003-022-03381-1)
Supplement: Supplementary file 2 — Supplementary Information [file 42003_2022_3381_MOESM2_ESM.pdf]

Supplementary Information

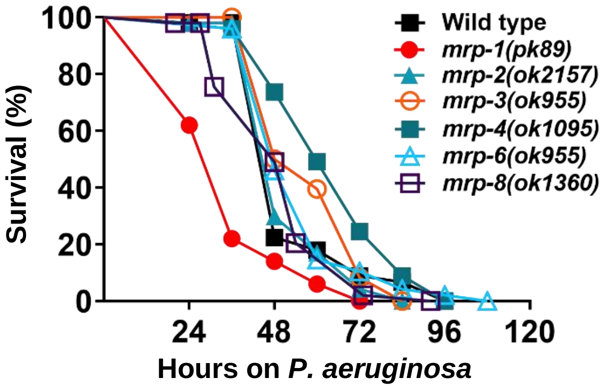

Supplementary Fig. 1. The *C. elegans* efflux pump MRP-1 is required for immunity against *P. aeruginosa*. Representative survival plot of wild-type and *mrp* mutant *C. elegans* strains exposed to a partial lawn of *P. aeruginosa*. The survival rate of *mrp-1(pk89)* mutant animals was compared to that of wild type using the log-rank (Mantel-Cox) test, and the differences were found to be significant, ****P < 0.0001. Assays were performed at 25°C, with 40–50 animals/assay.

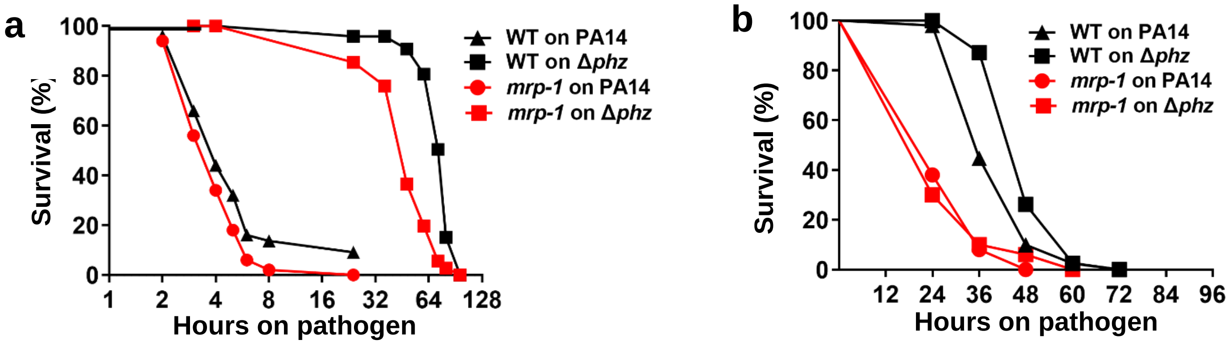

Supplementary Fig. 2. The susceptibility phenotype of *mrp-1* mutants is not determined by soluble phenazine toxins produced by *P. aeruginosa*. a) Representative survival plot of wild-type (WT) and *mrp-1(pk89)* mutant *C. elegans* animals exposed to a lawn of *P. aeruginosa* PA14 or a phenazine-deficient *P. aeruginosa* mutant strain (*Δphz*) under a fast-killing assay format. The survival rate of *mrp-1* animals exposed to PA14 was compared to that of wild type exposed to PA14 using the log-rank (Mantel-Cox) test, and the differences were found to be significant, *P = 0.0347. The survival rate of *mrp-1* animals exposed to Δ*phz* was compared to that of wild type exposed to Δ*phz* using the log-rank (Mantel-Cox) test, and the differences were found to be significant, ****P < 0.0001. **b)** Representative survival plot of wild-type (WT) and *mrp-1(pk89)* mutant *C. elegans* animals exposed to a lawn of *P. aeruginosa* or phenazine-deficient *P. aeruginosa* (*Δphz*) under a slow killing assay format. The survival rate of *mrp-1* animals exposed to PA14 was compared to that of wild type exposed to PA14 using the log-rank (Mantel-Cox) test, and the differences were found to be significant, ****P < 0.0001. The survival rate of *mrp-1* animals exposed to Δ*phz* was compared to that of wild type exposed to Δ*phz* using the log-rank (Mantel-Cox) test, and the differences were found to be significant, ****P < 0.0001. All the assays were performed at 25°C, with 40–50 animals/assay.

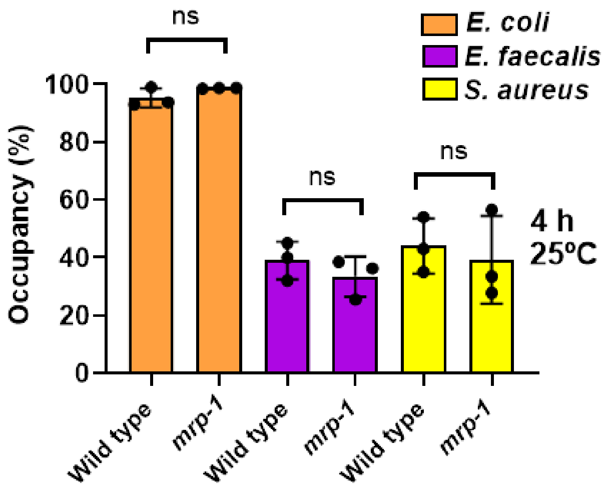

Supplementary Fig. 3. Lawn occupancy of wild-type and the *mrp-1(pk89)* mutant animals on a partial lawns of *E. faecalis*, *S. aureus*, or control *E. coli*. Each dot represents the average occupancy determined from three independent assays. Error bar represents the standard deviation. "ns'-nonsignificant according to a two-tailed t-test. All assays were performed at 25°C, with 40–50 animals/assay. The assay was performed as previously described[1].

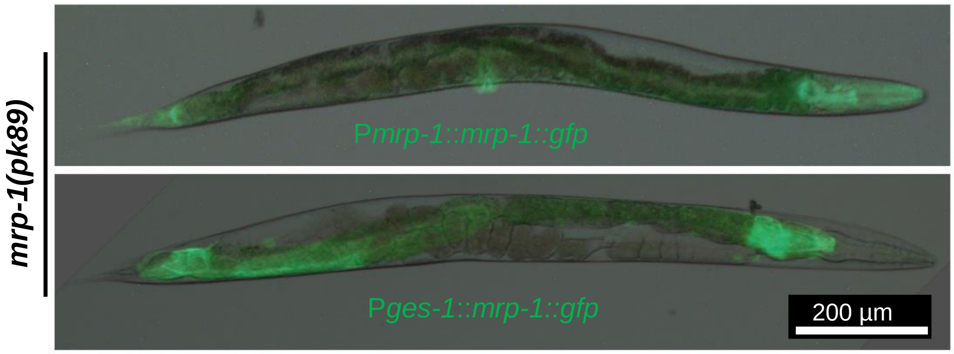

Supplementary Fig. 4. Representative photomicrographs of transgenic animals. Rescue of *mrp-1(pk89)* with translationally fused MRP-1::GFP expressed under the control of the P*mrp-1* homologous promoter or the P*ges-1* intestine-specific promoter.

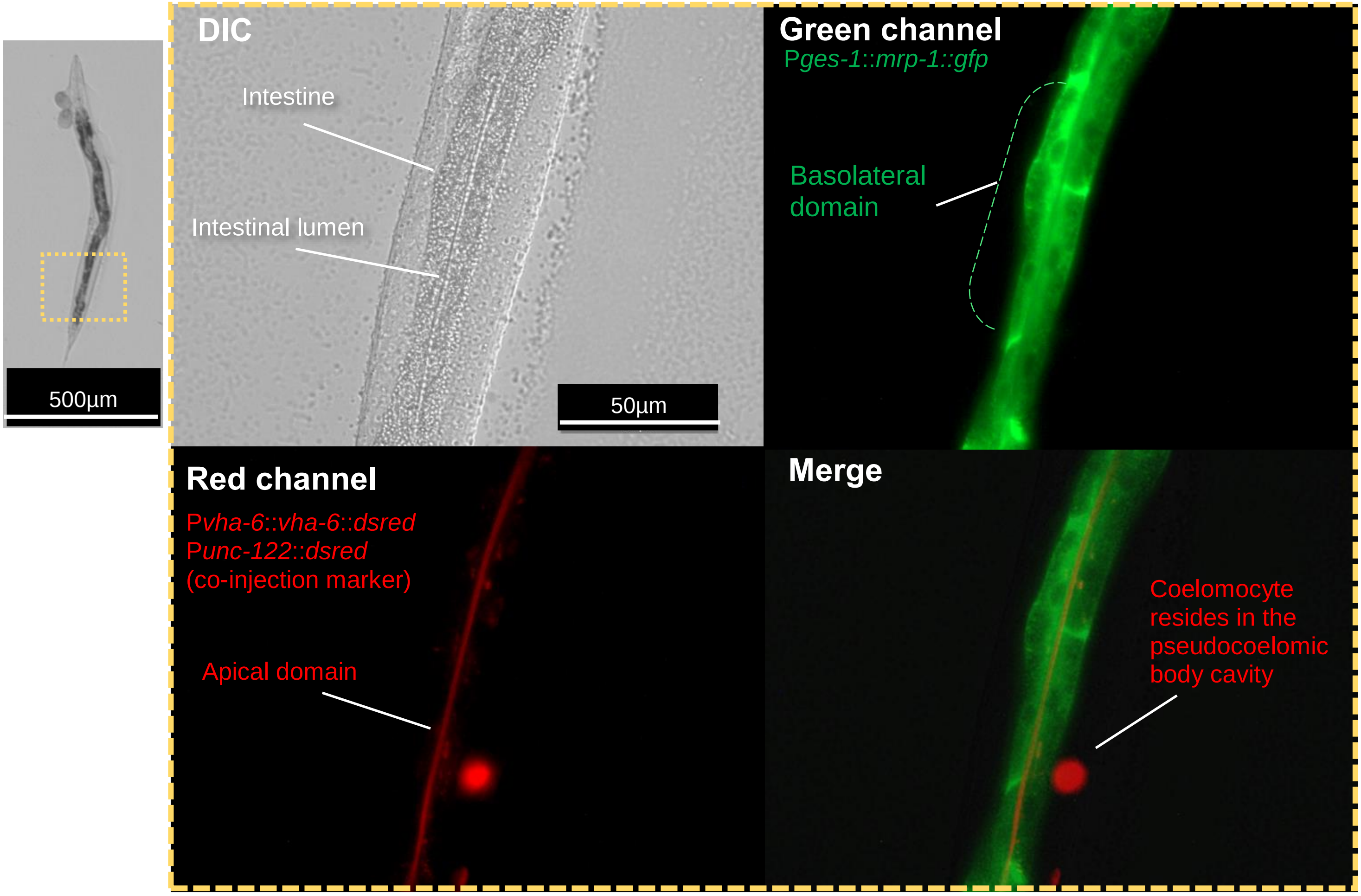

Supplementary Fig. 5. The translationally fused MRP-1::GFP protein does not co-localized with VHA-6::dsRED apical domain protein in the intestine. Representative photomicrograph of A transgenic animal generated via microinjection of pJL2 (pPD95.75_P*ges-1::mrp-1::gfp*), pJL6 (coel::RFP_P*vha-6::vha-6::dsred*) and coel::RFP (P*unc-122::dsred*) plasmid constructs.

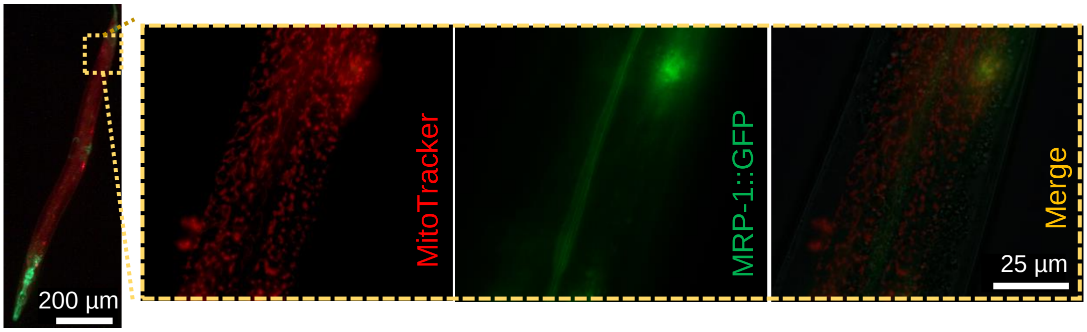

Supplementary Fig. 6. Representative photomicrographs of MRP-1::GFP transgenic animals. Larval stage-4 animals were exposed to 0.5 µl of MitoTracker Red dye (1 µg/µl concentration) prepared in 1 ml of M9 buffer. The animals were exposed for 5 h at 20 °C, followed by three washes with M9 buffer. Levamisole (0.25 mM) was used to immobilize the animals before microscopic observations.

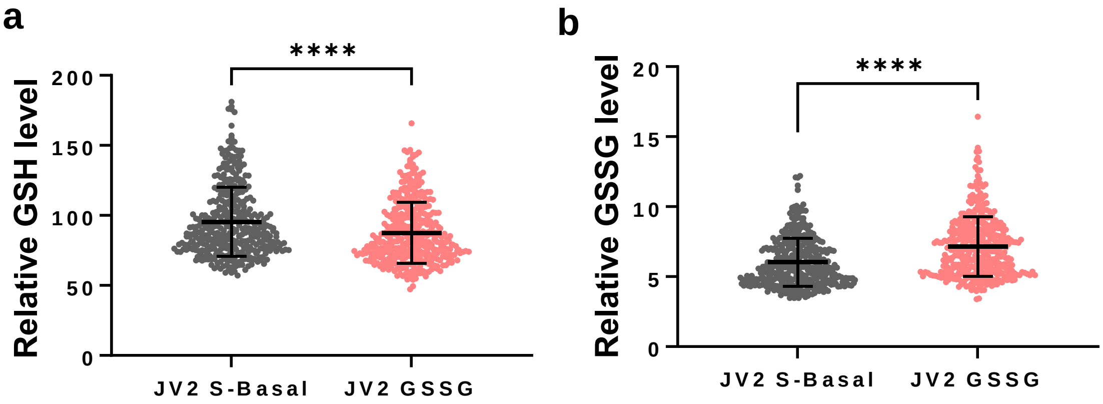

Supplementary Fig. 7. Relative GSSG levels in the JV2 roGFP sensor strain animals exposed to buffer or permeable methyl-GSSG. Synchronized young gravid animals were harvested and transferred to a snap cap-tube containing buffer only (S-basal medium, with 5% saturated *E. coli* OP50 broth culture as food source) or buffer supplemented with permeable GSSG methyl-ester at 25mM final concentration. The animals were exposed to the solution for 4-5 h at 25ºC with intermittent tapping and agitations. The animals were wash thrice with M9 buffer and then subjected to GSSG quantification followed by sorting and GSH quantification, using a COPAS Biosort flow cytometer. The relative levels of **a)** GSH and **b)** GSSG were determined by measuring the fluorescence signal intensity of $roGFP_{488nm}$ and $roGFP_{405nm}$, respectively. Each dot represents the fluorescence intensity of a single animal (area under the curve (AUC) x 1000 over time of flight (TOF)). Bars represent the mean ± SD, ****P ≤ 0.0001 according to the two-tailed t-test. '*' indicates a significant difference.

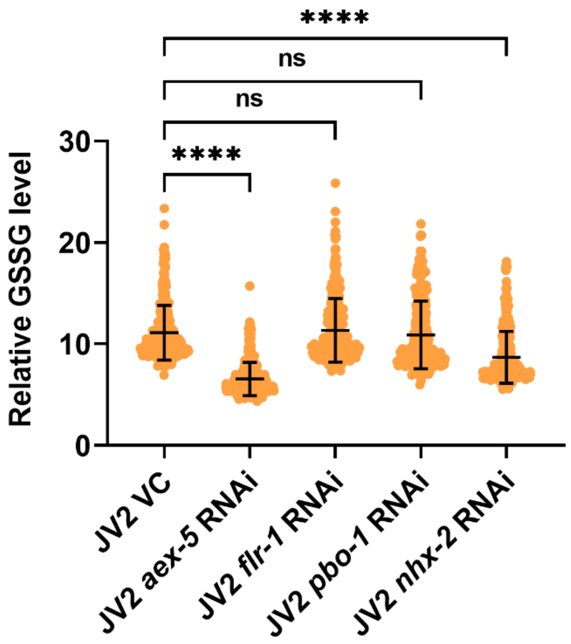

Supplementary Fig. 8. Quantification of GSSG levels of the JV2 roGFP sensor strain (JV2) upon the disruption of the defecation motor program. Relative GSSG levels of the sensor strain grown on vector control (VC) bacteria as well as RNAi bacteria that induce intestinal bloating. Synchronized L4-stage animals were transferred to RNAi plates and grown for 24 h. The animals were harvested and subjected to GSSG quantification using a COPAS Biosort flow cytometer. Each dot represents the fluorescence intensity of a single animal (area under the curve (AUC) x 1000 over time of flight (TOF)). Bars represent the mean ± SD, 'ns' indicates nonsignificant; '*' indicates a significant difference. ****$P \leq 0.0001$ via one-way ANOVA with Tukey's multiple comparison test, with 250–300 animals/assay.

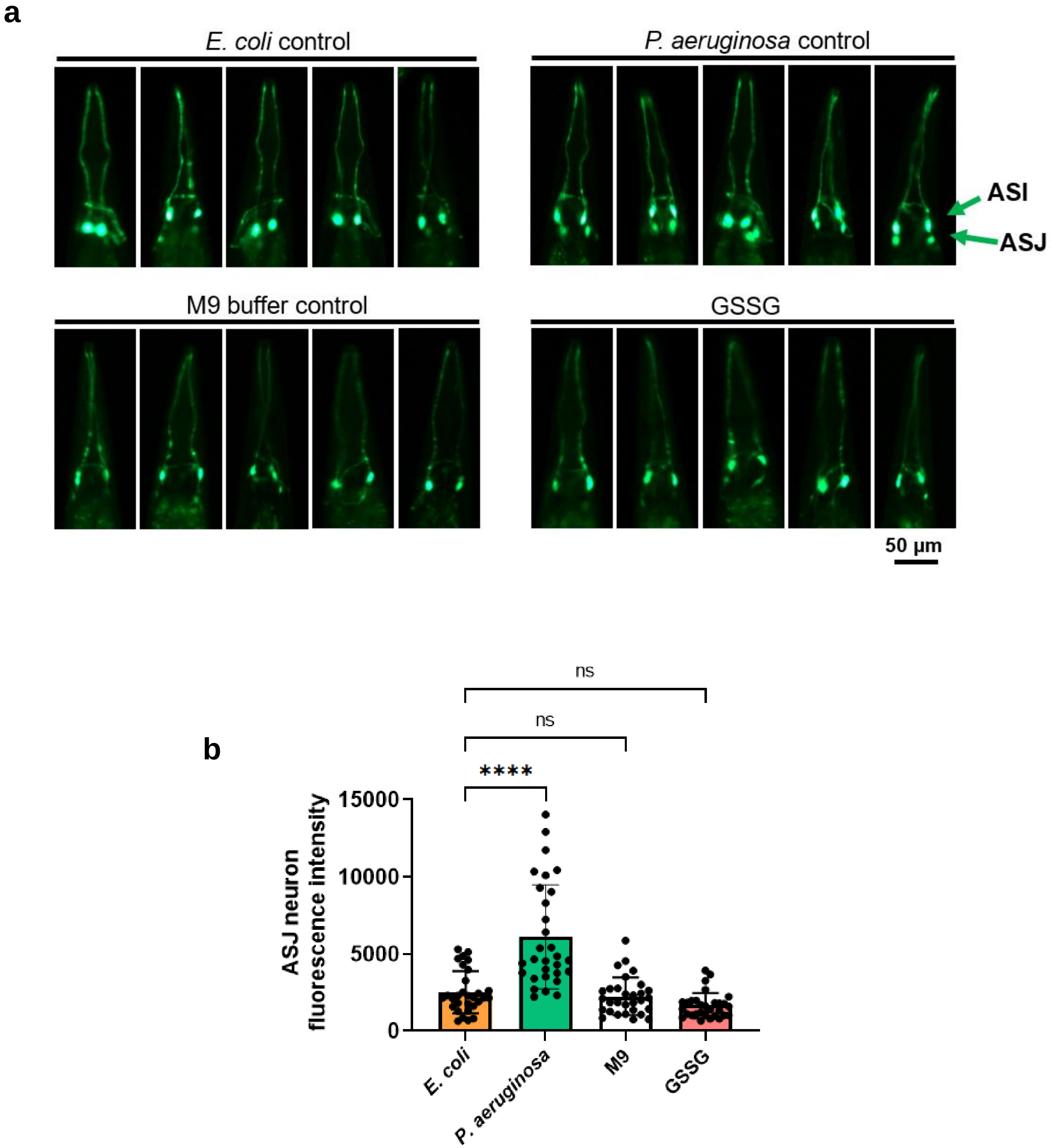

Supplementary Fig. 9. Effect of GSSG on the expression of *daf-7*. a) Representative photomicrographs of *daf-7p::gfp*-expressing animals exposed to *E. coli*, *P. aeruginosa*, M9 buffer control, or a 50 mM cell-permeable GSSG solution for 2 h at 25 °C. **b)** Quantification of fluorescence intensity in ASJ neurons. Each dot represents the fluorescence intensity of a single neuron (arbitrary units). Three independent experiments were performed, with 5 animals/assay. Bars represent the mean ± SD, ‘ns’ indicates nonsignificant; ‘*’ indicates a significant difference; ****P ≤ 0.0001 via ANOVA with Dunnett’s test.

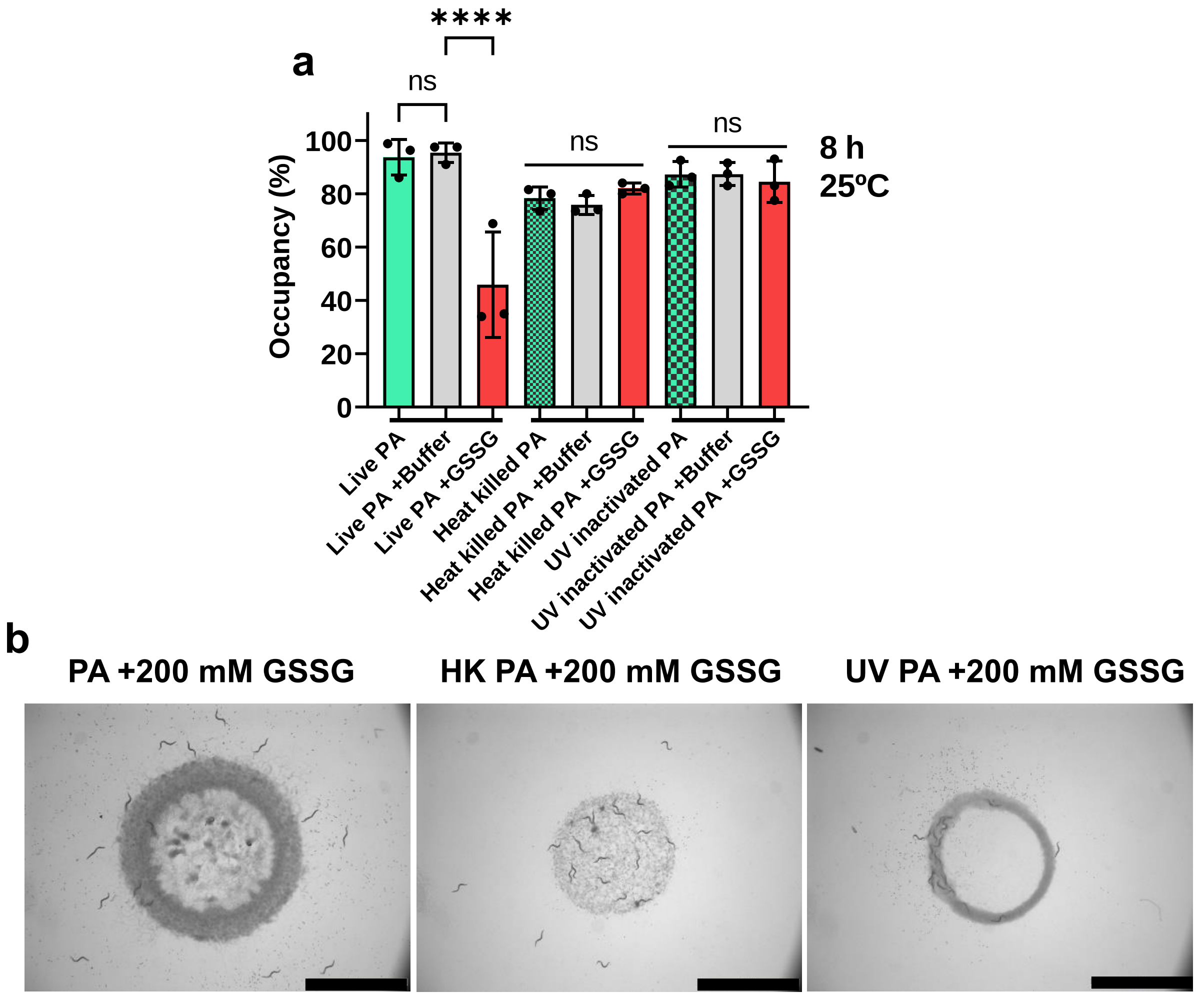

Supplementary Fig. 10. Live pathogenic interaction is required for the activity of GSSG molecule. a) Lawn occupancy of wild-type animals on a partial lawn of live (PA), heat killed (HK PA), or UV inactivated *P. aeruginosa* (UV PA), supplemented with or without 200mM GSSG at 8 h, 25ºC. Each dot represents the average occupancy determined from three independent assays performed at 25°C with 40–50 animals/assay. Bars represent the mean ± SD, 'ns' indicates nonsignificant; '*' indicates a significant difference; ****P ≤ 0.0001 according to one-way ANOVA followed by Tukey's multiple comparisons test. **b)** Representative images of the lawn occupancy of wild-type animals on partial lawns *P. aeruginosa* with varying lawn conditions. Images were acquired at 8 h. Scale bars are 5 mm. For the preparation of heat killed lawn, 16 h broth culture of PA14 was pelleted and resuspended to 40 times the concentration using the original liquid broth. The concentrated culture was heated on a dry bath at 100 ºC for 1 h. After cooling at room temperature, 10 µl of heat killed bacteria was seed on a 3.5 cm SK +antibiotics (kanamycin at 50 µg/ml, carbenicillin at 100 µg/ml) plate and allowed to set for overnight. For the preparation of the UV inactivated lawn, 16 h broth culture of PA14 was pelleted and resuspended to 20 times the concentration. Ten microliters of the concentrated culture were seeded on a 3.5 cm SK +antibiotics plate and allowed to air dry for 30 m. The seeded culture was then expose to UV radiation at 30000 µJ for 30 m (UV Stratalinker 1800, Stratagene). To ensure complete inactivation, the irradiation process was performed thrice. The effectiveness of the heat killing or UV inactivation of bacteria was determined by inoculating portion of the lawns into an LB broth. Sterility was confirmed when no bacterial growth was observed on the LB broth after overnight culture at 37ºC. For the supplementation, 5-10 µl of M9 buffer or 200 mM in M9 supplement solution was carefully place on the surface of the lawn, followed by air-drying for 1 h. Synchronized young gravid adult animals were washed with M9 containing 50µg/mL kanamycin and incubated at 20 ºC for 1 h and transferred to center of each lawn. The assay was performed at 25 ºC and the fraction of animals in or out of the lawn was scored.

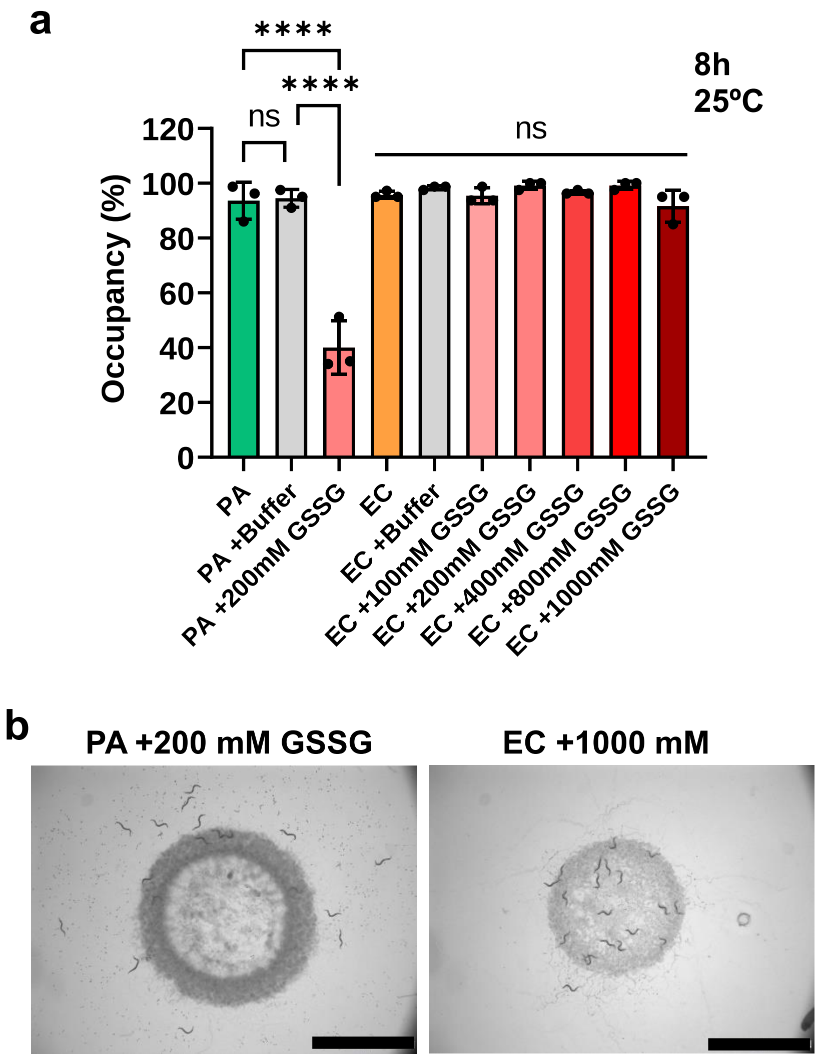

Supplementary Fig. 11. **GSSG supplementation is not sufficient to trigger lawn avoidance on nonpathogenic *E. coli***. **a)** Lawn occupancy of wild-type animals on a partial lawn of *P. aeruginosa* (PA) or *E. coli* (EC) supplemented with varying GSSG concentrations, at 8 h. Each dot represents the average occupancy determined from three independent assays performed at 25°C with 40–50 animals/assay. Bars represent the mean ± SD, 'ns' indicates nonsignificant; '*' indicates a significant difference; ****P ≤ 0.0001 according to one-way ANOVA followed by Tukey's multiple comparisons test. **b)** Representative images of the lawn occupancy of wild-type animals on partial lawns of *E. coli* or *P. aeruginosa* with GSSG supplementation. Images were taken at 8 h. Scale bars are 5 mm.

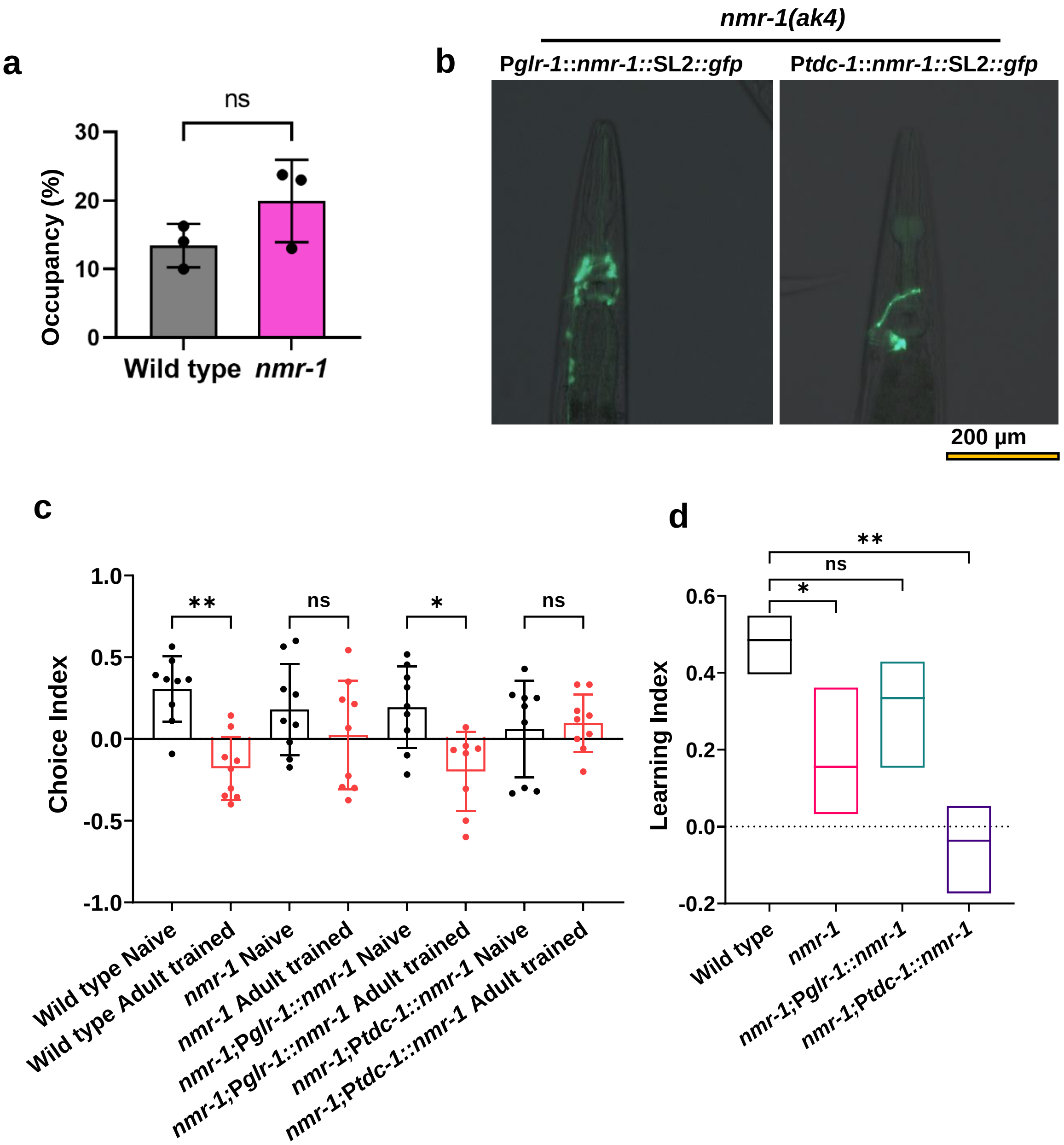

Supplementary Fig. 12. NMR-1 is required for behavioral immunity. a) Lawn occupancy of wild-type animals and *nmr-1(ak4)* mutant animals on a partial lawn of *P. aeruginosa* after 24 h. The bars represent the means (SDs) from three independent experiments. 'ns' indicates non-significant according to a two-tailed t-test, with 40–50 animals/assay. **b)** Representative fluorescent microscopy images of *nmr-1(ak4)* transgenic animals. **c)** Choice preference indexes of naïve and adult trained animals. Each dot represents a biological replicate from three independent experiments; each bar represents the mean ± SD of 100–200 1-day-old gravid adult animals/assay. **d)** Learning index after adult training on *P. aeruginosa* (PA14). Floating bars represent the mean and minimum-maximum range. P values were generated by ANOVA with Tukey's multiple comparisons test (B) and by ANOVA with Dunnett's correction (C); 'ns' indicates nonsignificant; '*' indicates a significant difference; $^{*}P \leq 0.05$; $^{**}P \leq 0.005$.

Supplementary References:

1. Filipowicz, A., Lalsiamthara, J. & Aballay, A. TRPM channels mediate learned pathogen avoidance following intestinal distention. *Elife* 10, (2021).
