# Supplementary material for: The gut efflux pump MRP-1 exports oxidized glutathione as a danger signal that stimulates behavioral immunity and aversive learning
Source: Commun Biol. 2022 May 5;5:422. doi: 10.1038/s42003-022-03381-1 (PMC9072357; doi:10.1038/s42003-022-03381-1)
Supplement: Supplementary file 5 — Reporting summary [file 42003_2022_3381_MOESM5_ESM.pdf]

nature portfolio

Corresponding author(s): Alejandro Aballay

Last updated by author(s): Apr 18, 2022

Reporting Summary

Nature Portfolio wishes to improve the reproducibility of the work that we publish. This form provides structure for consistency and transparency in reporting. For further information on Nature Portfolio policies, see our Editorial Policies and the Editorial Policy Checklist.

Statistics

For all statistical analyses, confirm that the following items are present in the figure legend, table legend, main text, or Methods section.

n/a	Confirmed	
☐	☒	The exact sample size (*n*) for each experimental group/condition, given as a discrete number and unit of measurement
☐	☒	A statement on whether measurements were taken from distinct samples or whether the same sample was measured repeatedly
☐	☒	The statistical test(s) used AND whether they are one- or two-sided<br>*Only common tests should be described solely by name; describe more complex techniques in the Methods section.*
☒	☐	A description of all covariates tested
☒	☐	A description of any assumptions or corrections, such as tests of normality and adjustment for multiple comparisons
☐	☒	A full description of the statistical parameters including central tendency (e.g. means) or other basic estimates (e.g. regression coefficient) AND variation (e.g. standard deviation) or associated estimates of uncertainty (e.g. confidence intervals)
☐	☒	For null hypothesis testing, the test statistic (e.g. *F*, *t*, *r*) with confidence intervals, effect sizes, degrees of freedom and *P* value noted<br>*Give P values as exact values whenever suitable.*
☒	☐	For Bayesian analysis, information on the choice of priors and Markov chain Monte Carlo settings
☒	☐	For hierarchical and complex designs, identification of the appropriate level for tests and full reporting of outcomes
☒	☐	Estimates of effect sizes (e.g. Cohen's *d*, Pearson's *r*), indicating how they were calculated

Our web collection on statistics for biologists contains articles on many of the points above.

Software and code

Policy information about availability of computer code

Data collection	No software was used.
Data analysis	GraphPad Prism 9.2.0.332

For manuscripts utilizing custom algorithms or software that are central to the research but not yet described in published literature, software must be made available to editors and reviewers. We strongly encourage code deposition in a community repository (e.g. GitHub). See the Nature Portfolio guidelines for submitting code & software for further information.

Data

Policy information about availability of data

All manuscripts must include a data availability statement. This statement should provide the following information, where applicable:

- Accession codes, unique identifiers, or web links for publicly available datasets
- A description of any restrictions on data availability
- For clinical datasets or third party data, please ensure that the statement adheres to our policy

All relevant data are within the manuscript and supplementary files.

Field-specific reporting

Please select the one below that is the best fit for your research. If you are not sure, read the appropriate sections before making your selection.

☒ Life sciences ☐ Behavioural & social sciences ☐ Ecological, evolutionary & environmental sciences

For a reference copy of the document with all sections, see nature.com/documents/nr-reporting-summary-flat.pdf

Life sciences study design

All studies must disclose on these points even when the disclosure is negative.

Sample size	No sample sizes were calculated. Isogenic population of Caenorhabditis elegans strains that show very little or no heterogeneity were used in this study. Also this study performed experiments in accordance with previously reported established protocols. The sample size used in the study is sufficient to draw a conclusion.
Data exclusions	No data were excluded from the analyses.
Replication	All attempt of replication were successful.
Randomization	No randomization process was performed. Isogenic population of Caenorhabditis elegans strains that show very little or no heterogeneity were used in this study.
Blinding	No blinding was performed during group allocation, or during data acquisition or analyses.

Reporting for specific materials, systems and methods

We require information from authors about some types of materials, experimental systems and methods used in many studies. Here, indicate whether each material, system or method listed is relevant to your study. If you are not sure if a list item applies to your research, read the appropriate section before selecting a response.

Materials & experimental systems

n/a	Involved in the study	
☒	☐	Antibodies
☒	☐	Eukaryotic cell lines
☒	☐	Palaeontology and archaeology
☐	☒	Animals and other organisms
☒	☐	Human research participants
☒	☐	Clinical data
☒	☐	Dual use research of concern

Methods

n/a	Involved in the study	
☒	☐	ChIP-seq
☐	☒	Flow cytometry
☒	☐	MRI-based neuroimaging

Animals and other organisms

Policy information about studies involving animals; ARRIVE guidelines recommended for reporting animal research

Laboratory animals	Model organism Caenorhabditis elegans, Sex-hermophrodites. Strains are obtained from the Caenorhabditis Genetics Center (CGC), Minneapolis, MN
Wild animals	The study did not involve wild animals
Field-collected samples	The study did not involve samples collected from the field.
Ethics oversight	No ethical approval or guidance was required, the study employed C. elegans model organism.

Note that full information on the approval of the study protocol must also be provided in the manuscript.

Flow Cytometry

Plots

Confirm that:

- [ ] The axis labels state the marker and fluorochrome used (e.g. CD4-FITC).
- [ ] The axis scales are clearly visible. Include numbers along axes only for bottom left plot of group (a 'group' is an analysis of identical markers).
- [ ] All plots are contour plots with outliers or pseudocolor plots.
- [x] A numerical value for number of cells or percentage (with statistics) is provided.

Methodology

Sample preparation

The roGFP-sensor strain JV2 jrIs2 [rpl-17p::Grx1-roGFP2 + unc-119(+)] nematode animals were synchronized on NGM plates containing E. coli. Young gravid-adult animals were harvested in M9 buffer and transferred to a tube containing S-basal buffer or permeable GSSG dissolved in S-basal; or a plate containing a lawn of P. aeruginosa (PA14) or control E. coli (OP50). After 4–5 h of incubation at 25°C, the animals were collected and washed three times with M9 buffer to remove bacteria and debris. The animals were then loaded on to the instrument.

Instrument

COPAS Biosort flow cytometer (Union Biometrica Inc.)

Software

FlowPilot version 1.6.1.0 (2009)

Cell population abundance

There is no issue with purity. Synchronized single population of the roGFP-sensor strain JV2 or JV2 crossed with mutant strain was used. Each dot/data point represents individual nematode animal.

Gating strategy

Young-gravid adult stage nematodes (roGFP sensor strain) were gated based on Extinction versus TOF (time of flight equivalent to length). The changes in fluorescence intensity due to different treatment versus control were measured and data were acquired.

- [ ] Tick this box to confirm that a figure exemplifying the gating strategy is provided in the Supplementary Information.
